# Supplementary material for: Responses of ticks to immersion in hot bathing water: Effect of surface type, water temperature, and soap on tick motor control
Source: PLoS One. 2021 Dec 17;16(12):e0261592. doi: 10.1371/journal.pone.0261592 (PMC8682875; doi:10.1371/journal.pone.0261592)
Supplement: Attachment — Submitted filename: Reviewer Response.pdf [file pone.0261592.s003.pdf]

Editor Comments:

1. Please ensure that your manuscript meets PLOS ONE's style requirements, including those for file naming. The PLOS ONE style templates can be found at

https://journals.plos.org/plosone/s/file?id=wjVg/PLOSOne_formatting_sample_main_body.pdf
https://journals.plos.org/plosone/s/file?id=ba62/PLOSOne_formatting_sample_title_authors_affiliations.pdf

We have reformatted our manuscript to following the style templates as requested.

2. In your manuscript, please provide the name of the butchery facility where you obtained the raw pig skin from.

The raw pig skin was obtained from an independent pig farmer in Minnesota. We have modified the sentence to read "…(b) raw pig skin from a farmer (A. Kozumplik) who raised and butchered the pig in Minnesota…" (lines 88 – 89)

3. Please amend your manuscript to include your abstract after the title page

The abstract has been added after the title page.

4. In your Data Availability statement, you have not specified where the minimal data set underlying the results described in your manuscript can be found. PLOS defines a study's minimal data set as the underlying data used to reach the conclusions drawn in the manuscript and any additional data required to replicate the reported study findings in their entirety. All PLOS journals require that the minimal data set be made fully available. For more information about our data policy, please see http://journals.plos.org/plosone/s/data-availability.

Upon re-submitting your revised manuscript, please upload your study's minimal underlying data set as either Supporting Information files or to a stable, public repository and include the relevant URLs, DOIs, or accession numbers within your revised cover letter. For a list of acceptable repositories, please see http://journals.plos.org/plosone/s/data-availability#loc-recommended-repositories. Any potentially identifying patient information must be fully anonymized.

We have uploaded our minimal data set as a Supporting Information file ("S1_Dataset.xlsx").

Reviewer 1: **The authors are appreciated for the written article, however there are numerous aspects to consider. They present an highly ambitious title by mentioning "avoiding tick-borne illnesses", however, no tick-borne agent is evaluated throughout the work, much less the diseases they cause. In many sections, colloquial language, and the absence of accurate data from their results prevail, which is extremely negative in the case of an experiment. At the same time, the authors mention the importance of the surface in relation to the attachment and subsequent detachment of the ticks, which is why I consider it necessary to test their hypotheses on human subjects (as they mentioned in lines 183-184).**

We thank the reviewer for their helpful feedback. We agree that "avoiding tick-borne illnesses" may be too ambitious as a title for the work that was done and have renamed the manuscript "Responses of ticks to immersion in hot bathing water: Effect of surface type, water temperature, and soap on tick motor control", and added a phrase in lines 63 – 64 "… various physical factors of bathing water…" to better reflect the intent of the paper.

While we agree with the reviewer that it is necessary to perform similar tests on human subjects, we see this as a potential next step since this conclusion was made based on the results of this current study (as rightfully pointed out by the reviewer in lines 203 – 204). Hence, we see our current work as what is needed to provide justification for obtaining approval to do such a human subjects study, while these results still provide valuable findings which demonstrate quantitatively the importance of surface type on tick attachment.

Other comments are listed below:

Line 60: What type of soap was used? It is not specified until line 99.

We have moved the description of the soap used from line 99 to the first mention of this soap on line 79 as rightfully suggested by the reviewer.

Line 95: Remove the underline.

We have removed the underline from the subheading.

Results- Experiments with Nymphs: It appeared to me that this entire section was written colloquially, without providing actual numbers for each described experience with ticks at each temperature change. The authors mention on several occasions "many" or "some" instead of indicating the actual number of individuals. Considering that this is an experiment, I think they should report all the results obtained, and not simply dictate "a few" or even "in rare instances".

The initial paragraph of this section was meant to give a qualitative description from observations of nymph movements and scenarios (e.g., bubble formation on the ventral side) during the experiment, which ranges widely from nymph to nymph to be quantified accurately. We felt that these qualitative observations are important to give readers a sense of the varied nymph reactions, with the most important quantitative results being the

number of nymphs staying attached to the surface after 3 minutes of immersion (Table 1) and a time distribution showing when the nymphs fell off (Fig 2). We have added a subheading on line 133: “Qualitative observation of nymph movement” to make clear our intention of this paragraph.

Line 144: Replace the % symbol and spell it.

We have replaced the % symbol with the word “percentage”.

Line 168-170: This sentence does not correspond to a scientific language.

We agree with the reviewer that the sentence “One of us watched a wild-acquired adult female dog tick on our skin in a hot bath at home walk to a shallower angle” is an anecdotal example rather than a scientific experiment, and hence have removed this line from the text.

Reviewer #2: Dear authors
I reviewed the manuscript entitled “Prospects for avoiding tick-borne illnesses through bathing: Effect of surface type, water temperature, and soap on tick attachment” by Schimpf and colleagues. The manuscript reports important data about the biology of ticks and the prevention of tick borne pathogens to humans. Overall, the study was well planned and the text is well written, with novel aspects about these vectors. Therefore, the text deserves to be published on PLOS One as is it.

We thank the reviewer for the positive review!

Reviewer #3: The article (PONE-D-21-27474), entitled “Prospects for avoiding tick-borne illnesses through bathing: Effect of surface type, water temperature, and soap on tick attachment”, provides potential future perspectives against the spread of tick-borne illness, discussing experimental data on tick attachment phenomena, according to different kind of environmental/external conditions. Overall, methods are technically sound (including laboratory procedures and statistical tools) and presented in a clear way, as well as results and their implications widely consistent. I do believe this manuscript deserves publication in PLOS ONE, pending minor revisions which are listed below.

Materials and methods
Line 49: dog tick Dermacentor variabilis is a kind of definition too general, considering other tick species which are recognized under this common name (e.g., Ixodes canisuga, Rhipicephalus sanguinesu sensu lato, etc…). Please, replace with “American dog tick”, which is the common name more largely employed to define Dermacentor variabilis.
Line 50: members of the family Ixodidae.
Line 52-53: “Ticks were tested after they had stopped excreting visible dark residue of blood ingested at their previous stage”. Here could be useful to report the timeline or

at least an average of the number of days after which the tick specimens stopped to excreting visible dark residue of blood.
Line 54: family Acaridae.
Line 66: “acrylic platform held 45° above …”. Here the Authors have to clarify which degree (i.e., Celsius or Fahrenheit, etc…). Of course, they refer to °C but, anyway, reporting this information is absolutely necessary.
Line 95: Please, remove underline from “and temperature”.
Results
Table 2: In the caption of Table 2, the Authors provide a specific definition for “Soon” and Never, but quotation marks are reported only for soon, not for never. Please, add quotation marks also for the word never.
Line 226: Please, use the abbreviation B. burgdorferi, since you already define the scientific name in extenso at the line 221.
Line 238: Please, use features instead of facets.

Thank you for these suggestions; all changes have been made in the text (highlighted in yellow).

Regarding the timeline which the tick specimens stopped excreting visible dark residue of blood, the amount of time here varied a lot within the experiment as a whole, but it was at least one week after blood-meal excretion had stopped in all cases. After careful consideration, we decided that this point was not crucial to the manuscript and hence have deleted this sentence from the paper.

Regarding the acrylic platform, 45° refers to the angle at which the platform is held to the horizontal and not a temperature. We have rephrased this to read “…held at an angle of 45 degrees above horizontal…” instead.
