# Supplementary material for: Responses of ticks to immersion in hot bathing water: Effect of surface type, water temperature, and soap on tick motor control
Source: PLoS One. 2021 Dec 17;16(12):e0261592. doi: 10.1371/journal.pone.0261592 (PMC8682875; doi:10.1371/journal.pone.0261592)
Supplement: Attachment — Submitted filename: Review PONE-D-21-27474.docx [file pone.0261592.s002.docx]

Prospects for avoiding tick-borne illnesses through bathing: Effect of surface type, water temperature, and soap on tick attachment

The authors are appreciated for the written article, however there are numerous aspects to consider. They present an highly ambitious title by mentioning "avoiding tick-borne illnesses", however, no tick-borne agent is evaluated throughout the work, much less the diseases they cause. In many sections, colloquial language, and the absence of accurate data from their results prevail, which is extremely negative in the case of an experiment. At the same time, the authors mention the importance of the surface in relation to the attachment and subsequent detachment of the ticks, which is why I consider it necessary to test their hypotheses on human subjects (as they mentioned in lines 183-184).

Other comments are listed below:

Line 60: What type of soap was used? It is not specified until line 99.

Line 95: Remove the underline.

Results- Experiments with Nymphs: It appeared to me that this entire section was written colloquially, without providing actual numbers for each described experience with ticks at each temperature change. The authors mention on several occasions "many" or “some” instead of indicating the actual number of individuals. Considering that this is an experiment, I think they should report all the results obtained, and not simply dictate "a few" or even “in rare instances”.

Line 144: Replace the % symbol and spell it.

Line 168-170: This sentence does not correspond to a scientific language.
